# Humoral immunity to SARS-CoV-2 vaccination in haemodialysis patients (Response to: Humoral and cellular immunity to SARS-CoV-2 vaccination in renal transplant versus dialysis patients: A prospective, multicenter observational study using mRNA-1273 or BNT162b2 mRNA vaccine.)

**DOI:** 10.1016/j.lanepe.2021.100237

**Published:** 2021-10-25

**Authors:** Sandra Hasmann, Michael Paal, Louise Füeßl, Michael Fischereder, Ulf Schönermarck

**Affiliations:** a Department of Medicine IV, University Hospital, LMU Munich; b Institute of Laboratory Medicine, University Hospital, LMU Munich

In their prospective multicentre study Stumpf et al. report on the humoral and cellular immunity to SARS-CoV-2 vaccination in the so far largest cohort of dialysis patients [1]. mRNA-based COVID-19 vaccines elicit an antibody response in 80-96% of the dialysis population (Table 1), which is substantially higher compared to hepatitis B or influenza vaccinations.

**Table 1:** Comparison of SARS-CoV-2 S antibody response rate* and median antibody⁎⁎ titre after vaccination between haemodialysis⁎⁎⁎ patients and healthy controls⁎⁎⁎⁎ using the median and the interquartile range.

	HD patients SARS-CoV-2-Antibody	HC	p-value#	test method	cut-off / unit
Danthu et al., Clin J Am Soc Nephrol 2021	RR 81% (N=78) Ab titre 278 (83-526)	RR 100% (N= 7) Ab titre 1082 (735-1662)	<0,001	LIAISON SARS-CoV-2 TrimericS IgG (DiaSorin)	> 13 AU/ml
Espi et al., Kidney Int 2021	RR 82% (N=106) Ab titre 176	RR 100% (N=30) Significant higher titre, but no median reported	<0,001	Maglumi® SARS-CoV-2 S-RBD IgG test (Snibe Diagnostic)	> 1 AU/ml
Grupper et al., Clin J Am Soc Nephrol 2021	RR 96% (N=56) Ab titre 2900 (1128-5651)	100% (N=95) Ab titre 7401 (3687-15471)	<0,001	SARS-CoV-2 IgG II (Abbott)	> 50 AU/ml
Jahn et al., vaccines 2021	RR 93% (N=72) Ab titre 366,5 (89.6-606)	100% (N=16) Ab titre 800 (520-800)	<0,001	LIAISON® SARS-CoV-2 TrimericS IgG (Diasorin)	AU/ml
Simon et al., Nephrol Dial Transplant 2021	RR 91% (N=81) Ab titre 171	RR 100% (N=80) Ab titre 2500	<0,001	Elecsys Anti-SARS-CoV-2 S (Roche)	> 0,4 U/ml
Paal et al., Clin Kidney J 2021	RR 96.6% (N=179) Ab titre 253.5 (64.2 - 679)	RR 97.1%(N=70)## Ab titre 1756 (971.5- 2436.5)	<0,001	Elecsys Anti-SARS-CoV-2 S (Roche)	≥ 0,8 U/ml
Stumpf et al., Lancet Reg Health Eur 2021	RR 95.5% (N=1256)	RR 98.5% (N=144)		SARS-CoV-2 specific IgG- or IgA-antibody reactions (Euroimmun)	
Yanay et al., Kidney Int 2021	RR 90% (N=160) Ab titre 116.5 (66-160)	RR 100% (N=132) Ab titre 176.5 (142-235)	<0,001	LIAISON SARS-CoV-2 TrimericS IgG (DiaSorin)	AU/ml

⁎ response rate (RR), defined as Antibody titre above the cut-off of the assay.

⁎⁎ antibody (Ab).

⁎⁎⁎ haemodialysis (HD).

⁎⁎⁎⁎ healthy controls (HC).

# p-value comparing the median Ab titre, if reported.

## cohort of non dialysis patients.

However, when looking in detail at the quantity of the antibody titre their humoral response is significantly lower than in health care workers or non-dialysis patients (Table 1). More than half of the haemodialysis patients develop a titre below the lowest Anti-SARS-CoV-2 S titre in the control group [2]. In contrast, haemodialysis patients with a prior infection mount a substantially higher antibody response [3] comparable to non-dialysis individuals [2,4].

In addition to this apparent diminished antibody response in dialysis patients, concerns also remain about faster waning of antibody levels after vaccination in this group, as is described after natural infection [5]. Given that neutralizing antibody levels have been shown to be highly predictive of immune protection from symptomatic SARS-CoV-2 infection [6], dialysis patients may benefit from regular antibody testing and intensified vaccine schedules. Therefore further studies should establish antibody thresholds predictive of protection from severe disease, and clarify the role and timing of booster vaccinations in this group.

## Author contributions

Sandra Hasmann and Ulf Schönermarck designed the letter; all authors contributed significantly to the content of the letter and have accepted the final version.

## Compliance with ethics guidelines

This article is based on previously conducted studies and does not contain any new studies with human participants or animals performed by any of the authors.

## Declaration of interests

The authors have nothing to disclose.
